# The eye lens: age-related trends and individual variations in refractive index and shape parameters

**DOI:** 10.18632/oncotarget.5762

**Published:** 2015-09-27

**Authors:** Barbara Pierscionek, Mehdi Bahrami, Masato Hoshino, Kentaro Uesugi, Justyn Regini, Naoto Yagi

**Affiliations:** ^1^ Faculty of Science, Engineering and Computing, Kingston University London, Kingston-upon-Thames, United Kingdom; ^2^ Japan Synchrotron Radiation Research Institute (SPring-8), Sayo, Hyogo, Japan; ^3^ School of Vision Sciences, Cardiff University, Cardiff, United Kingdom

**Keywords:** ageing, eye lens, refractive index gradients, growth, size, Gerotarget

## Abstract

The eye lens grows throughout life by cell accrual on its surface and can change shape to adjust the focussing power of the eye. Varying concentrations of proteins in successive cell layers create a refractive index gradient. The continued growth of the lens and age-related changes in proteins render it less able to alter shape with loss of capacity by the end of the sixth decade of life. Growth and protein ageing alter the refractive index but as accurate measurement of this parameter is difficult, the nature of such alterations remains uncertain. The most accurate method to date for measuring refractive index in intact lenses has been developed at the SPring-8 synchrotron. The technique, based on Talbot interferometry, has an X-ray source and was used to measure refractive index in sixty-six human lenses, aged from 16 to 91 years. Height and width were measured for forty-five lenses. Refractive index contours show decentration in some older lenses but individual variations mask age-related trends. Refractive index profiles along the optic axis have relatively flat central sections with distinct micro-fluctuations and a steep gradient in the cortex but do not exhibit an age-related trend. The refractive index profiles in the equatorial aspect show statistical significance with age, particularly for lenses below the age of sixty that had capacity to alter shape *in vivo*. The maximum refractive index in the lens centre decreases slightly with age with considerable scatter in the data and there are age-related variations in sagittal thickness and equatorial height.

## INTRODUCTION

The lens of the eye is a key optical element that, together with the cornea, refracts light to the retina. Unlike the cornea, the lens has the capacity to alter ocular focus so as to enable clarity of vision for objects over a range of distances. This functional capacity, termed accommodation, which decreases progressively with age leading to a loss of ability to focus on near objects, is achieved by the lens changing its shape under forces mediated by the contraction and relaxation of the ciliary muscle. Accommodative capacity is effectively lost by the sixth decade of life for, though the rates can vary in individuals based on a number of internal and external factors [1, 2], by the end of the sixth decade when atrophy of the ciliary muscle occurs [1] lenses have no ability to accommodate. Refractive power for distant objects is not affected or altered with age. The growth of the lens continues throughout life by accrual of layers of fibre cells over existing tissue without concomitant cellular loss creating a lamellar structure with the oldest cells in the central layers and the most recently synthesised cells on the lens surface. This renders the lens an ideal tissue for the study of ageing and pathologies of ageing given that chronic systemic conditions of protein folding such as diabetes have manifestations in the eye lens [3]. A suggested link between Alzheimer's disease and cataract [4] was not supported by findings from more recent studies [5, 6], perhaps indicating that a systemic condition needs to be present for a longer term than is Alzheimer's disease before any associated signs are seen in the lens.

The refracting function of the lens is determined by its optical power which in turn depends on its external shape as well as on the refractive index of its medium. The latter is linearly related to protein concentration [7]. The cytoplasmic proteins, the crystallins, contained within the cell layers of the lens, are distributed in different concentrations throughout the lens in such a way as to form a concentration, and therefore a refractive index, gradient [8, 9, 10]. The property of an index gradient provides improved image quality for the eye and, given that the growth mode creates a chronological lamellar formation, it would not be implausible to consider that there may be distinct patterns of change in the refractive index gradient that are indicative of ageing and could be differentiated from pathological processes. If such changes could be detected in the lens they could ultimately be used clinically to develop a direct *in vivo* means of measuring rates of ageing.

The lens contains features that appear similar to the rings of a tree and are seen when looking into a living eye using a biomicroscope [11]. These are called zones of discontinuity [11–13] and they have been studied to determine whether their presence is a manifestation of specific episodes linked to development and growth [11]. Such links were confirmed [11] and whilst the study, which looked *in vivo* lenses from 100 subjects and found that zones broaden and appear denser with age, clear ageing trends related to the medium of the lens in the central or nuclear region could not be ascertained [11]. Recent findings, using a new highly accurate method of measuring the refractive index, have linked this to the zones of discontinuity providing the first evidence of causal factors [13]. The functional reason for these zones, if indeed there is one, and the link between the refractive index profile and ageing remains unknown. A principal reason for this is the difficulty in measuring the refractive index. This is a highly sensitive measure and any change in hydration of tissue will alter the result. Ideally this should be conducted using a direct, non-invasive method on the intact lens that can determine subtle variations in refractive index in any plane of the lens. To understand the changes that occur with age and that underpin age-related cataract formation, requires a method that can measure refractive index in older lenses in which the processes of opacification that may lead to age-related cataract, have commenced. The combined use of synchrotron radiation and advanced interferometric methods [13, 14] has provided a means of measuring refractive index that can detect subtle changes as well as those that arise because of individual variations [15, 16]. The technique has been used in this study to measure the refractive index in a large cohort of human lenses in order to investigate age-related trends and whether individual variations exist. These findings have implications for considering a more personalised approach to the design of implant lenses used for treatment of cataract. Novel implant designs that incorporate a gradient index lens and take into account individual variations in optical properties of the lens will provide an improved image quality that is matched to the visual demands of the individual.

## RESULTS

**Figure 1:**
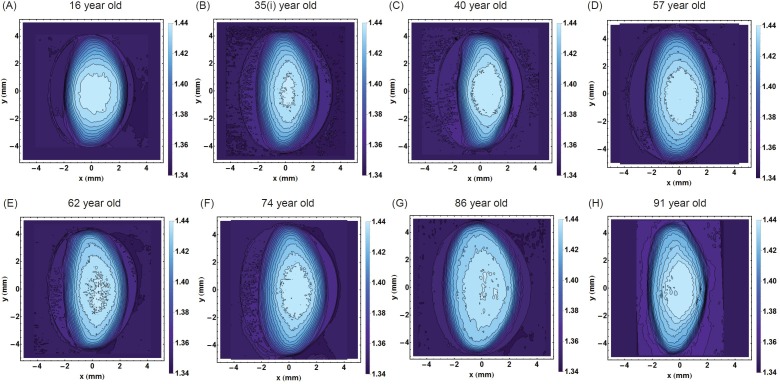
Iso-indicial contours of refractive index in the sagittal plane for eight representative human lenses aged **A.** 16 years, **B.** 35 years, **C.** 40 years, **D.** 57 years, **E.** 62 years, **F.** 74 years, **G.** 86 years, **H.** 91 years. The bisector of the sagittal plane is the optic axis which is pertinent to vision. [The 35 year old lens is labelled 35**I**. to distinguish it from the 35 year old lens in Figure 8].

The refractive index contours in the sagittal plane and along the optic axis from eight representative lenses spanning the age range studied are shown in Figure 1. Each contour has a constant refractive index and the magnitude of refractive index increases with progression into the centre. The youngest lens (Figure 1A) has an approximately round central contour and, with movement away from the centre, the contours become more elliptical. In this lens the contours are approximately centred around the same point. In all the other lenses (Figure 1B to 1H), contours are elliptical and, apart from the 35 year old lens (Figure 1B), the contours in the other lenses (Figure 1C to 1H) are not centred. The decentration is most obvious in the older lenses, aged 86 and 91 years old (Figure 1G and 1H) but does not show a clear ageing trend: the 57 year old lens has greater contour centration than the 40 year old lens. The size and smoothness of the central contour does not appear to show any age-related trends. The 35 year old lens in Figure 1B) has a relatively small and irregular central profile, the 57 year old lens (Figure 1D) has a larger smoother central contour and the 62 year old lens (Figure 1E) has a central contour with numerous inflexions and breaks.

**Figure 2:**
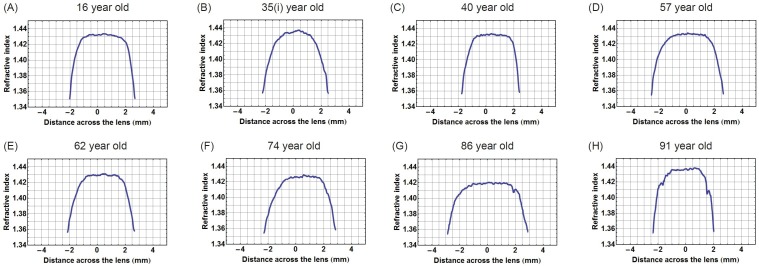
Refractive index variations plotted in the sagittal plane against distance along the optic axis for eight representative human lenses aged **A.** 16 years, **B.** 35 years, **C.** 40 years, **D.** 57 years, **E.** 62 years, **F.** 74 years, **G.** 86 years, **H.** 91 years.

Figure 2 shows the corresponding refractive index plotted along the optic axis in the eight lenses, the contours of which were shown in Figure 1. The central sections that cover two-thirds of the profiles have an almost constant region with small fluctuations and steep gradients on either side. In the 35 year old lens (Figure 2B) the central section is the least flat and in the older lenses, aged 86 and 91 years (Figure 2G and 2H), there are sharp inflections or kinks in the gradient section; these are particularly evident in the oldest lens (Figure 2H).

**Figure 3:**
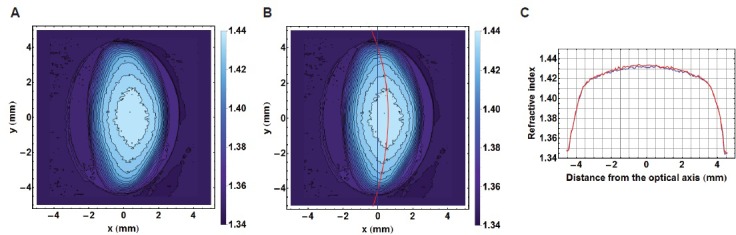
**A.** Iso-indicial contours in the sagittal plane of a 52 year-old human lens; **B.** the true equatorial plane running through the centre of each contour and depicted by the red curve shown with the equatorial height (blue line); **C.** The refractive index profiles along the equatorial plane (in red) and the equatorial height (in blue).

**Figure 4:**
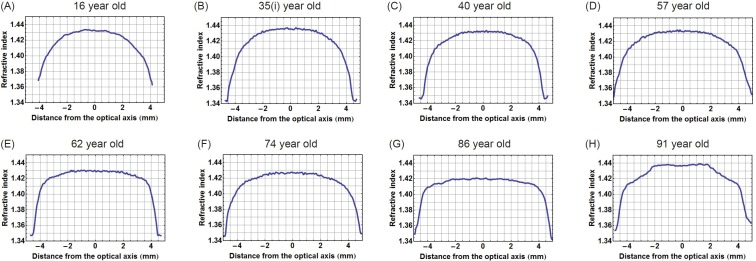
Refractive index variations plotted in the equatorial plane against distance from the optic axis for eight representative human lenses aged **A.** 16 years, **B.** 35 years, **C.** 40 years, **D.** 57 years, **E.** 62 years, **F.** 74 years, **G.** 86 years, **H.** 91 years.

In view of the decentralisation of the refractive index contours (as shown in Figure 3A), plotting refractive index in the equatorial plane requires ensuring that each contour is bisected along the longest axis. In lenses where contours are decentralised, this provides a curved equatorial plane (red curve in Figure 3B). Taking the equatorial plane as a straight line that represents the height of the lens (blue line in Figure 3B), as is commonly assumed [10] would not correctly intercept the centres of all contours nor is this a true representation of the biological equatorial plane. Figure 3C) compares the refractive index profiles plotted against the equatorial plane and along the straight line that runs the length of the lens. There is little difference in the steep gradient sections between the two profiles with slight discrepancies seen in the central portion; the true equatorial refractive index profile (red curve) having a slightly greater magnitude and fewer fluctuations than the profile plotted against the straight line through the length of the lens (blue line). Figure 4 shows the true equatorial profiles for the eight lenses seen in Figure 1. In the youngest lens the profile most closely approximates a parabolic function (Figure 4A). With age there is a general trend to steepening of the peripheral sections and a flattening and widening of the central part of the profile. In the very oldest lenses, 86 and 91 year old (Figure 4G and 4H respectively) the central regions are almost constant and the distinctions between the peripheral gradient and the central plateau are sharply defined. In the very oldest lens there are three such distinctions, the gradient section rising steeply in the extreme periphery, then there is an abrupt change in gradient to one with a less steep incline and an almost flat central region (Figure 4H).

The refractive index distribution can be approximated by a power law equation:

n = n_max +_ (n_min_ - n_max_) × r^2g^

where n is the index along the optic axis or equatorial plane

n_max_ is the maximum of index at the lens centre

n_min_ is the minimum of index at the lens surface

r is the normalised distance from the lens centre and

g is the power exponent.

**Figure 5:**
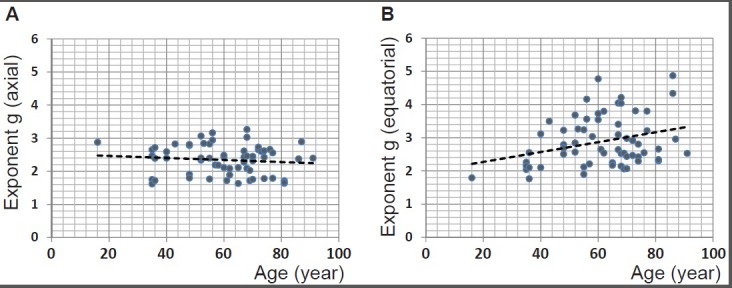
**A.** The power law exponents from fits to sagittal refractive index profiles for 63 human lenses aged between 16 to 91 years plotted against age (years): linear regression gives y = −0.0031 × age + 2.5261 (R^2^= −0.004, *p* = 0.389); **B.** the power law exponents from fits to equatorial refractive index profiles for 63 human lenses aged between 16 to 91 years plotted against age (years): linear regression gives y = 0.0148 × age + 1.9753 (R^2^= 0.081, *p* = 0.013).

The exponent g for the refractive index profiles along the optic axis and equatorial plane are plotted against age in Figure 5A and 5B respectively for 63 lenses. (For three of the samples the refractive index contours in the sagittal plane exhibited irregular shifts such that accurate power law fits could not be deduced). There is no statistically significant relationship between g along the optic axis and age (*p* = 0.389). Plotting the exponent from the equatorial profile fits against age (Figure 5B) shows a trend that is statistically significant (*p* = 0.013). When this data set is divided into younger (below 60 years of age) and older cohorts (60 years of age and older) to distinguish between lenses with and without accommodative capacity, statistical significance is only found for the younger cohort (*p* = 0.003).

**Figure 6:**
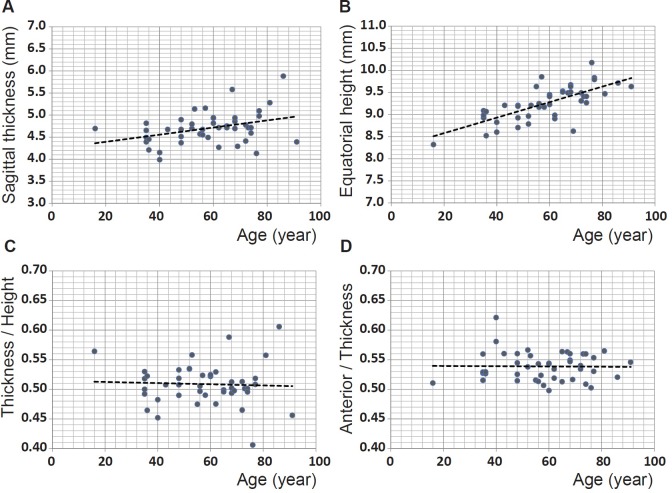
**A.** Sagittal thickness (mm) for 45 human lenses aged between 16 and 91 years plotted against age (years): linear regression gives y = 0.0081 × age + 4.2318 (R^2^=0.110, *p* = 0.0149); **B.** equatorial height (mm) for 45 human lenses aged between 16 and 91 years plotted against age (years): linear regression gives: y = 0.0176 × age + 8.2274 (R^2^=0.510, *p* =<<0.0001); **C.** ratio between sagittal thickness and equatorial height for 45 human lenses aged between 16 and 91 years plotted against age (years): linear regression gives y = −0.0001 × age + 0.5144 (R^2^=−0.02, *p* = 0.766); **D.** ratio between the anterior section of the lens, from the anterior pole to the equatorial plane, to the total thickness for 45 human lenses aged between 16 and 91 years plotted against age (years): linear regression gives: y = −0.0001 × age + 0.5399 (R^2^=−0.023, *p* = 0.923).

The other parameter that contributes to refractive power is lens shape. In forty-five of the samples, the outer shape had a sufficiently sharp image to enable collection of accurate data. The sagittal thickness (the distance along the optic axis from anterior to posterior pole) and the equatorial height of these lenses are shown in Figure 6A and 6B respectively. The thickness and height show statistically significant increases with age (*p* = 0.0149 and *p* << 0.0001 respectively). When the cohort is divided into ages below and above the age of 60 years, the sagittal thickness is not statistically significant for either cohort (figures not shown): *p* = 0.157 and *p* = 0.463 for the younger and older groups respectively. Conversely, the equatorial height is statistically significant for both groups (figures not shown): *p* = 0.0008 and *p* = 0.032 for the younger and older sets respectively. The ratio of sagittal thickness to equatorial height is plotted against age in Figure 6C and the ratio of anterior part of the sagittal thickness (from anterior pole to the equatorial plane) to the total sagittal thickness is plotted against age in Figure 6D. Neither show any age-related trends nor are statistically significant (*p* = 0.766 for the lens thickness to height ratio against age in Figure 6C and *p* = 0.922 for the anterior thickness to total thickness ratio against age in Figure 6D) indicating that there is no age-related variation in shape.

**Figure 7:**
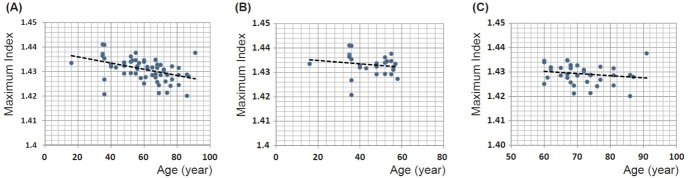
**A.** Maximum refractive index for 66 human lenses aged between 16 to 91 years old plotted against age (years): linear regression gives y = −0.0001 × age + 1.4385 (R^2^=0.185, *p* = 0.00018); **B.** maximum refractive index for the cohort of 28 lenses aged between 16 to 58 years plotted against age (years): linear regression gives y = −0.0001 × age + 1.4363 (R^2^=−0.011, *p* = 0.405); **C.** Maximum refractive index for the cohort of 38 lenses aged between 60 to 91 years old plotted against age (years): linear regression gives y = −0.0001 × age + 1.4355 (R^2^= 0.006, *p* = 0.278).

The maximum refractive index from all lenses investigated is plotted against age in Figure 7A. The magnitude of maximum refractive index varies from 1.420 to 1.441 and there appears to be a slight decrease with age that is statistically significant (*p* << 0.001). A linear equation fitted to the data gives the relationship: maximum refractive index = −0.0001 × age (years) + 1.4385. On closer perusal there are wide individual variations in lenses from the fourth decade (aged 35 and 36 years); there is only a single lens younger than this. The trend to decrease with age appears from Figure 7A to be most evident in lenses aged 60 years and above. When the data are divided into two age cohorts, above and below the age of 60, there is no statistical significance with *p* = 0.405 and 0.277 for the younger or older cohorts respectively (Figure 7B and 7C).

**Figure 8:**
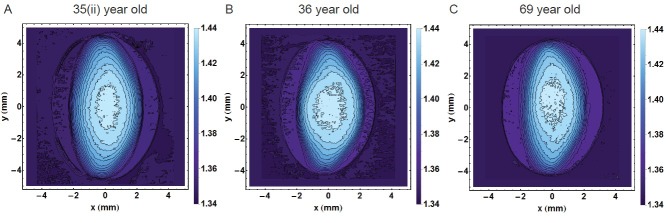
Refractive index contours in the sagittal plane for **A.** 35 year old lens; **B.** 36 year old lens; **C.** 69 year old lens showing the range of individual variations. (The 35 year old lens is labelled 35(ii) to distinguish it from the 35 year old lens in Figure 1).

The high degree of scatter in many of the figures indicates individual variations that mask age-related trends. Such individual variations are seen clearly in Figure 8 which shows the sagittal contours of three lenses aged 35, 36 and 69 years. Whilst all three lenses differ in shape and contours, the outer shape and the contours of refractive index in the 35 year old lens are more akin to those of the 69 year old lens than to those of the 36 year old lens.

## DISCUSSION

Ageing is a natural physiological process that leads to some well-defined changes in the body, such as loss of skin elasticity, bone resorption, retardation in mechanisms of repair and is a major risk factor of many chronic and acute diseases. It is also a process that continues to occupy a wide range of research fields as the more specific and the subtle manifestations of ageing on the structure and function of various body systems require far greater understanding that current knowledge permits. The eye lens, because of its growth mode as a continual accrual of new cells over existing tissue without concomitant tissue loss, retains a chronological record of its growth, development and ageing processes. Hence, it offers a unique opportunity to study ageing from the physiological and pathological perspective. The lens can be clearly viewed within the living eye to discern changes in its structure; its function can be measured clinically and can manifest pathological changes that occur with systemic disease [3].

Since ageing is a time based variable, a simplistic assumption and one used often in the absence of other information, is that age in years represents a uniform rate of ageing. This assumption does not always apply. The rate of ageing can vary as it is influenced by genetic and external factors, such as lifestyle. With regard to the accommodative function of the eye lens, the assumption that age-related loss is linked to age in years is considered to be justified as accommodation decreases with age at a rate that is considered to be well-established and has even been predicted by a simple mathematical equation [17]. The loss of accommodative capacity starts to manifest as difficulty with focussing on near objects, generally around the fifth decade of life [1, 2] with total loss of capacity by the end of the sixth decade [1, 18, 19]. At this point the lens cannot alter its shape to allow the eye to focus on objects at close range and reading glasses are the most common treatment. Whilst function may be lost at a well-established rate, the causes of such loss are multifactorial including changes in shape and geometry and alterations in material/structural properties. The contributions of each factor are difficult to determine and may vary from person to person [19].

The other age-related change in the lens is the gradual loss of transmittance and transparency [18,20] which, when advanced to such a stage that insufficient light can traverse the lens to reach the retina, is termed cataract. These changes involve the crystallin proteins which are the basic structural entities of the lens. Their concentrations are linked to the refractive index by a linear relation [7] and their varied concentration across the lens creates the gradient of refractive index (reviewed in [10]). How the gradient index in the lens alters with age has been the subject of a number of studies involving human and animal lenses and a variety of different methods have been used to measure this parameter (reviewed in [10]). Because the refractive index depends on the concentration of proteins, any change in tissue properties that could alter this, most notably dehydration or protein loss when the tissue is sliced, will change the refractive index. Ultimately, the method of measurement should be non-invasive and conducted on an intact lens. Previous investigations on whole lenses have used ray tracing, which requires mathematical calculations applicable to a symmetrical shape [8, 21]. In the lens such symmetry is only found in the equatorial plane which is perpendicular to the plane that contains the optic axis ie the sagittal plane. In order to determine refractive index along the optic axis, mathematical transposition of the equatorial refractive index to the sagittal plane and assumptions to be made about the refractive index contours are required [8, 21]. The method is most applicable and accurate for spherical or near spherical lenses such as are found in fish [22–27] or rodents [28, 29] but less so for the human lens which ideally requires a method that can measure the refractive index directly in any plane. The other non-invasive method that has been used is Magnetic Resonance Imaging (MRI) [30, 31]. This method detects free water from which refractive index is calculated but neglects the protein bound water that contributes to refractive index and overlooks the fact that the proportions of free and bound water alter with age [32]. Other indirect methodologies necessitate a number of assumptions, some untested (reviewed by [10]). The recent advance in measuring refractive index in the lens is based on Talbot interferometry with a synchrotron X-ray source [13, 14] allowing such measurements to be made on intact lenses in any plane and it has produced the most detailed profiles to date [15,16]. These measurements have indicated that the refractive index gradient is not smooth but has distinct irregularities and fluctuations [15, 16] which supports a finding reported previously in the African cichlid fish lenses [25].

The results from this study show that the shape and refractive index vary in different lenses and that the changes are not all related to age as individual variations mask ageing trends. As the lens grows throughout life there is a reasonable expectation that size may be related to age. Given that growth of lens layers begins on the anterior surface, changes in the geometry between the lens and zonule suggest that growth may led to an increased asymmetry [19, 33]. From the changes to lens proteins with age, indicated by a greater proportion of insoluble protein extracted from older compared to younger lenses [34], an alteration in the refractive index may also be expected. The size of lenses has been reported to increase with age [35–37] and this relationship is most evident with respect to lens height (Figure 6B). Though thickness plotted against age shows more scatter (Figure 6A), age-related changes in both height and thickness are statistically significant. The ratios between thickness and height (Figure 6C) as well as between the anterior and posterior sections of the thickness (Figure 6D) have no correlation with age indicating that shape variations are not age-related. Previous work on excised human lenses using MRI and scanning laser measurements reported a statistically significant increase with age in lens equatorial diameter but not in thickness [30].

It is worth considering that when the lens is removed from the eye, it is no longer under the tension that is imparted, *in vivo*, from the ciliary muscle and zonule that hold the lens in place and that mediate the forces that change lens shape. Lenses, pliable enough to accommodate *in vivo*, will therefore plausibly be expected to take up an accommodated form when removed from the eye and released from tension of the ciliary muscle and zonule. It is not possible to say exactly how accommodated an excised lens will be without seeing how each lens shape changes within the eye across the accommodative range of which that eye is capable. Accommodation is used to greater or lesser extents depending on lifestyle of an individual and how much time is spent on viewing close objects compared to sighting in the distance. It is also influenced by the refractive error and whether or not an individual is short or long-sighted. The latter tend, in younger years if not corrected with spectacles, to require a greater accommodative effort than the short-sighted or those without refractive error in order to compensate for the lack of refractive power of the eye. It is not possible to ascertain lifestyle factors or refractive error from a donor tissue but it may be surmised that lenses, capable of shape change, could be in different states of accommodation when removed from the eye and that these effects may be significant enough to mask trends related to ageing. The findings in this study are consistent with previous studies on *in vitro* lenses that show individual variations superimposed on ageing trends [30, 35, 39, 40]. Rosen et al [40] from a study of thirty-seven lenses, aged 20-99, found the ratio of equatorial diameter to sagittal thickness to be around 2 to 2.1 which corresponds approximately to the value of around 0.51 for the inverse ratio of thickness to diameter found in this study (Figure 6C). Rosen et al [40] reported that in spite of scatter, the ratio was statistically significant. This is not supported by the findings of this study. Lenses beyond the sixth decade of life, have no accommodative capacity [1]. Therefore on removal from the eye, these lenses, not able to alter their curvature or shape, should be in the same unaccommodated state as they are in the eye and can be compared without the compounding uncertainty of accommodative state. The equatorial height shows statistical significance with age for this older cohort only.

Individual variations are also seen in the relative positions of the refractive index contours. Whilst there may appear to be a tendency for the inner contours to shift posteriorly with age relative to the external shape (Figure 1), this is not a gradual progression: the 40 year old lens (Figure 1C) has a greater asymmetry in refractive index contour position than the 57 year old lens (Figure 1D). The more apparent change is in the shapes of the refractive index contours with the more circular inner contours becoming more elliptical with age. While this is most evident in the 16 and 40 year old lenses (Figure 1A and 1C respectively) compared to older lenses, some individual variations are found here as well: the 57 year old lens (Figure 1D) has a more circular inner contour than the 35 year old sample (Figure 1B) which defies the ageing trend. The degree of individual variation is further evidenced in examples given in Figure 8. The displacement of the contours may indicate something about the effects of stresses and strains on the lens during the action of accommodation and that whilst accommodative ability may be age-related, accommodative effort *in vivo* varies with refractive state of the eye and lifestyle demands on the visual system. This may manifest in the individual variations seen here. Refractive index contours measured using MRI [30] reported circular central contours in young lenses (aged 7 and 20 years) similar to those seen in the 16 year old lens in this work. Older lenses in the previous study (from age 50), however, had no central contours but an almost constant refractive index value over a greater part of the lens [30] which is not supported by these findings. This is likely to be because the previous work showed index contour increments of 0.01 [30] whilst in this study the contour increments were of 0.005.

Decentration of the contours means that the true equatorial plane is not along a straight line that runs along the height of the lens but follows a curved path. Higher order power law functions have been used in previous studies as these represent the simplest, yet closest mathematical representations of the gradient index profile [30, 41–46]. Power law fits can be adapted to account for variations in lens shape, such as toricity, and provide an accurate means of calculating optical power as well as aberrations of the lens and can thereby be used to predict image quality of the eye [46]. Power law functions were fitted to the refractive index profiles along curved equatorial planes. Plotting the exponents against age shows that in this plane the exponent variation with age is statistically significant (Figure 5B). Ageing trends can also be seen from the refractive index profiles in the equatorial plane (Figure 4). The shapes of the refractive index profiles along the optic axis, as determined by power law fits, do not have any relationship with age (Figure 5A). Previous work using MRI on twenty excised lenses, aged from 7 to 82 years, reported that, qualitatively, power law fits with low values of the exponent corresponded to younger lenses whilst those with higher values were more applicable to older lenses [30]. It was reported that with age the refractive index profiles in both the sagittal and equatorial planes became flatter in the central region [30]. This study found that some older lenses had a flatter central region and steeper cortical gradients but that this trend was only seen in the equatorial and not in the sagittal plane. The interesting finding in this study was that in the oldest lens, aged 91 years, there are three distinct regions in the equatorial refractive index profile with a narrower portion of the profile representing the constant refractive index section (Figure 4H). Kinks are apparent in the cortical sections of the profile along the optic axis of this lens (Figure 2H). A single kink appears in one side of the cortical profile along the optic axis of the 86 year old lens (Figure 2G). The kinks in the profile correspond to localised areas of higher density (particularly apparent in Figure 1H) and could be indicative of small opacities. The contours in the 91 year old lens progress further into the centre with a considerably smaller central contour corresponding to the narrower plateau portion (Figure 4H). This was not found previously [30]. It should be noted that in the earlier work [30], the profiles shown were from only two lenses at the extreme ends of the ageing scale (7 and 82 years of age) and it was not clear whether uneven nature and lack of smoothness of the profiles represented real discontinuities in refractive index or artifacts of the measurement [30]. The older lens was shown slightly tilted and interestingly had a thinner sagittal width than the younger lens which suggests that the sagittal profile may not have been taken exactly on axis [30] as in this study. The interferometric method used in this study, with 900 projections scanned from each lens, provides a far more accurate measure of refractive index than that deduced from measurements of free water using MRI [30]. Small fluctuations in refractive index in the plateau regions of the profile do not contribute to refraction. They produce a very low degree of back-scatter which, when used in an optical simulation, were found to create features similar to the zones of discontinuity [15].

The apparent age-related decrease in the maximum refractive index (Figure 7A) that is found in the very central part of the lens needs to be carefully considered. Clinical studies have reported an increase in curvature with age particularly of the anterior surface of the lens [37, 47–49] although wide individual variations were noted. The distorting effects of the optics of the eye when recording images through the cornea and lens have been recognised [37, 49, 50, 51]. Nevertheless, the original studies [47, 48] prompted the question of why the eye does not become more short-sighted with age as the more curved the lens the greater refractive power it provides and this should render the eye more short-sighted. Since the only other parameter of refractive power is the refractive index, it was suggested that this should decrease with age to compensate for an increase in curvature with age [52]. An apparent decrease in central refractive index from MRI studies was reported as evidence in support of this theory [31] but as MRI measures free water, which increases with age [32], the apparent decrease in central refractive index can been explained as not having accounted for bound water [10]. Subsequent studies using MRI have not found a statistically significant change in central refractive index with age [30]. The causal factors of a decrease in refractive index would need to be considered: this would require a decrease in protein content or an increase in water, neither of which have been found [53] nor could be explained given the physiology and anatomy of the lens [10]. What has been suggested is that the gradient section of the refractive index profile in the cortex of the lens alters its steepness with age, with no change in the central, plateau region, or nucleus, offsetting the change in curvature without necessitating any alteration in protein or water content [41]. This theory was subsequently supported by modelling studies [43] and experimental work [30]. These results are also consistent with earlier measurements of protein concentration gradients using microradiography on thin sections of frozen human lenses that found central regions of lenses had relatively constant protein concentrations and that the cortical gradients altered in steepness with age [54]. No significant changes in protein concentration with age were found [54]. This method is sufficiently precise to be able to detect sub-capsular cataract [55] and sub-capsular zones of discontinuity in the human lens [56]. Subsequent studies using Raman microspectroscopy found that water gradients had a similar form to protein concentration and refractive index profiles [57, 58] but water content was found to be lower in localised regions of the cortex, close to the cortico-nuclear area, than in the nucleus with these dips more prominent in profiles along the visual axis than in those along the equator [57]. It was suggested that this region of the lens could be predisposed to earlier cataract onset [57]. The steepening of the cortical gradients with age in the equatorial region found in this study were also reported for water profiles [58] and may be indicative of the slowing down of the growth rate of the lens in the equatorial region [58]. Interestingly, water content measured in the nuclear region of eighteen lenses appeared to increase with age [58]. For lenses below the age of fifty there was no apparent trend with age with very wide variations found for lenses from the fourth and fifth decades, which accords with the findings of this study [58].

When the lens changes its power to alter focus of the eye the refractive index must also undergo redistribution. From clinical observations, only the nucleus alters under the process of accommodation, expanding when the lens becomes more curved and contracting as it is stretched [59,60]. Whether or not this alters the magnitude of the refractive index in the lens centre is not known. An expansion of the plateau part of the lens in the nucleus and a concomitant steepening of the gradient in the cortex so that it rises to a higher magnitude in the plateau region of the profile would contribute to an increase in refractive power; light bends when there is a change in refractive index and the steeper that change, the greater the refraction. In such case, the statistically significant age-related decrease in this maximum refractive index that is seen when considering the whole cohort of lenses (Figure 7A), may be explained by the fact that younger lenses are in more accommodated states and that it may be the process of accommodation and not ageing *per se* that is reflected in the trend. In order to determine whether there is indeed an age-related change in maximum refractive index, lenses should be in the same state of accommodation. Taking the cohort aged 60 and older, there was no significant change in the maximum refractive index with age. A regression analysis of maximum refractive index against sagittal thickness and equatorial height gives no statistical significance with either shape parameter, although the relationship between refractive index and equatorial height borders on significance (*p* = 0.053 maximum refractive index against equatorial height; *p* = 0.798 maximum refractive index against sagittal thickness).

The lenses used in this study were frozen and thawed as it was not possible to obtain fresh samples in Japan necessitating transportation of tissues from the UK. The effects of freezing in both dry ice and at −20°C and subsequent thawing on protein structural order in bovine lenses was investigated in a previous study using X-ray diffraction [61]. Neither freezing method was found to have an effect on spacing or structure [61].

The lenses in this study did not show any disruptions to the refractive index profiles that may be suggestive of cataract. It has been reported from a study on donor lenses from an eye bank in Amsterdam that only around 20% of lenses from the eighth decade of life and under 9% from the ninth decade were clear [62]. These were early cataracts [62] which this is consistent with the fact that these lenses had not been removed and replaced by implants. Although ageing is a major risk factor for cataract, the lenses used in this study were those without opacification; any samples with early opacities were the subject of a separate study.

This study highlights the contribution of individual variations in the lens that can mask ageing trends. The continued growth and consequent increase in lens size with age is statistically significant and in the equatorial plane age-related changes to the profile of refractive index are statistically significant particularly for younger lenses. However, there is no correlation between age and the fit to the profile of refractive index along the optic axis. Decreases in the central refractive index with age show significance for the whole cohort but not when considering younger and older cohorts separately. The relevance of these trends to understanding the contribution of growth, ageing and lens dynamics is most important for the design of implant lenses and to future considerations of personalised implants that are able to alter to mimic the accommodative capacity in the eye. The incorporation of a gradient refractive index into implant design will improve image quality as the gradient provides a better control of aberrations than would a homogenous index lens [46]. This prospect is becoming more feasible as technologies for making gradient index lenses based on the biological lens are now being developed [63]. In time such designs could be adapted to produce a range of different gradient profiles that can be used to calculate the refractive power of the lens and ultimately to have this matched to the optical demands of the individual.

## MATERIALS AND METHODS

Human eyeballs (66 samples aged from 16 to 91 years), from which the corneal discs had been removed for transplantation, were obtained from the Bristol Eye Bank (UK). Samples were transported in dry ice to the SPring-8 synchrotron radiation facility in Japan and were thawed gently before measurement, after which lenses were removed from eyeballs and set in a physiologically balanced agarose gel within a specially designed sample holder [15,16]. The refractive index in each lens was measured using the X-ray phase contrast tomography based on the X-ray Talbot grating interferometer [13,14] constructed at the bending magnet beamline BL20B2 at SPring-8 [15,16]. Briefly, the instrument utilizes a 25 keV X-ray beam that passes through a Si(111) double crystal monochromator and two transmission gratings: a phase grating (G1) made of tantalum and an absorption grating (G2) made of gold with pattern thicknesses 2.1 μm and 16.6 μm respectively. Both gratings have a pitch of 10 μm and a pattern size area of 25mm (width) by 25mm (height). The inclination angle of G2 is 45 degrees. Moiré fringe patterns produced by X-ray passage through the sample and gratings are transmitted to an X-ray imaging detector that incorporates a beam monitor and a scientific CMOS detector (ORCA Flash 4.0. Hamamatsu Photonics). The field of view is limited by the width of the gratings. For phase retrieval, G2 was shifted with a Piezo stage with a 5-step ‘on-the-fly’ fringe-scan method [15]. Images from differential phase shifts were obtained and merged to produce the phase shift image. Calibration of the phase shift was made against five known-density solutions: water and salt solutions of 1.006 g/cm^3^, 1.051 g/cm^3^, 1.110 g/cm^3^, 1.143 g/cm^3^ [16]. Theoretical values were compared to experimentally obtained phase shift values per pixel and found to be linear over the concentration range tested which extend well beyond the range corresponding to the refractive index magnitudes in human lenses [16]. Phase shift values per pixel were converted to X-ray refractive index difference from saline to determine protein density which is related linearly to refractive index in the lens (the mathematical equations are described in detail in Hoshino et al [16]). Measurement on each sample took 50 min providing 900 projection images for each lens. Repeat measurements were conducted on four lenses and found to be reproducible. The maximum error in measurement is calculated from the experimental standard deviation in the phase shift, which was typically around 0.00033 radian. From calculations given in [16], this gives a maximum error in refractive index of + 0.0005. Cubic splines were used to connect points to create isoindicial contours of 0.005 increments in refractive index. Mathematica computational software v9 was used to visualize refractive index data and to fit power law functions to refractive index profiles. ANOVA regression analysis was performed with Microsoft Excel 2010. Ethical approval for this study was obtained from the National Health Service (NHS) committee (Oxford, UK).
